# A Design Method for Rectangular Waveguide-Typed Microwave Devices Based on a Novel Origami Process

**DOI:** 10.3390/ma16247625

**Published:** 2023-12-13

**Authors:** Yipeng Sun, Chuyuan Gao, Lijun Chen, Lei Han

**Affiliations:** 1 Key Laboratory of MEMS of the Ministry of Education, Southeast University, Nanjing 210096, China; syp2099@163.com (Y.S.); 220211686@seu.edu.cn (C.G.); chenlijun@seu.edu.cn (L.C.); 2 Nanjing Research Institute of Electronics Technology, Nanjing 210039, China

**Keywords:** origami, microwave device, rectangular waveguide, coupler, power divider, polystyrene, polymers, selective absorption

## Abstract

A novel design method based on a novel origami process that can create a solid structure swiftly and at a low cost is presented for rectangular waveguide-type microwave devices in this paper. A planar structure was fabricated by the lamination and laser cutting of polystyrene membranes and aluminum foils and was converted into a solid structure via origami in accordance with the selective absorption of infrared light. A rectangular waveguide, a rectangular waveguide-type coupler, and a power divider based on an origami structure with a multi-layer structure and a single-layer structure were fabricated and tested, demonstrating easy assembly and good microwave performance. The measured results of the rectangular waveguide indicated that the insertion loss was superior to −0.9 dB. Meanwhile, the results of the coupler showed that the coupling degree increased from −12.8 dB to −8.9 dB in the range of 11.0 GHz to 12.0 GHz. Correspondingly, the prepared power divider demonstrated that the return loss dwindled from −8.9 dB to −11.3 dB and that the insertion loss of one output port was approximate to that of the remaining one, varying between −3.5 dB and −5.2 dB in the range of 10.5 GHz to 11.5 GHz—verifying the effectiveness of the origami-based design method.

## 1. Introduction

Benefiting from the advantages of a large power capacity and low attenuation, rectangular waveguide-type microwave devices are playing an increasingly important role in modern communication systems. Traditional rectangular waveguide devices are manufactured by the method of laser beam machining with computer numerical control [1,2]. However, the manufacture of solid structures in rectangular waveguide-type microwave devices is laborious, time-consuming, and costly, with poor control of the process’ accuracy [3]. To solve these problems, 3D-printing technology has been introduced, which can realize complex solid structures accurately without the need for machine operations and molds [4,5,6,7,8]. Nevertheless, the stacking processing method of 3D-printing technology leads to the disadvantage of slow speeds in processing solid structures, while it can complete the machining of planar structures swiftly. Meanwhile, corresponding 3D modeling is required, which can be complicated for sophisticated solid structures [9]. To make matters worse, the appearance of printed samples can be too rough for applications in mm-wavebands [10]. Origami is a traditional manual art form that forms a variety of three-dimensional structures by folding two-dimensional planar structures. It originated in China and spread to Japan and other countries during the Tang Dynasty. With the development of science and technology, origami can be combined with practical applications to solve complex difficulties encountered in engineering, providing new solutions to engineering problems that cannot be solved by traditional processes. In order to give full play to the fast processing speeds of planar structures, origami technology that can convert planar structures into solid structures has been taken into consideration and widely investigated [11,12,13,14]. The main methods driving origami structures include differential pressure drives, mechanical drives, and active material drives [15]. Polymers and composites activated by uniform heating or localized heating have been extensively studied to achieve the origami method. In 2007, Suzuki et al. adopted creases composed of materials or material stacking to achieve folding and achieved creases through multiple manufacturing steps [16]. The material of the crease component is different from the substrate material, and only the crease component can respond to external stimuli. In 2011, Liu et al. harnessed the selective absorption of infrared light to locally heat a Shrinky-Dinks membrane through ink-defined hinges, leading to self-folding [17]. In 2014, Tolley et al. took advantage of shape memory composites that were composed of one layer of shape memory polymer (SMP) sandwiched between two structural layers to realize self-folding under the conditions of uniform heating in an oven [18]. In 2015, Mu et al. proposed to realize self-folding based on active graphene materials by using the different absorption/release abilities of graphene oxide and graphene to water under different light conditions [19]. In 2018, Zhang et al. proposed the use of a sandwich structure to realize the transformation from a two-dimensional planar structure to a three-dimensional structure. As a scaffold, the elastomer expands and contracts through the hydration/dehydration of the middle-part hydrogel, and realizes the bending/restoration of the whole structure under the constraint of the scaffold [20]. In 2022, Jin et al. presented a soft pneumatic actuator inspired by Kresling origami, which can twist and shrink via vacuum control [21]. Previous research has concentrated on the use of origami and its applications in the fields of robots [22,23], foldable aircraft [24,25], intelligent electronic components [26,27,28], and so on. In 2014, Hayes et al. presented self-folding origami microstrip antennas; they transformed a conventional microstrip transmission line into a monopole antenna [29]. In 2019, Nauroze et al. realized thermally actuated origami-inspired multilayer Mirua frequency-selective surfaces using a polyester-based substrate [30]. In 2020, Kaddour et al. presented a novel origami reflectarray unit-cell on the basis of the Miura–Ori pattern in order to obtain efficient folding and a high packing efficiency [31]. In 2023, Han et al. realized a 3D-shaped microstrip microwave device using an origami structure [32]. However, the existing research on origami-based microwave devices has only focused on improving microwave performance, such as reducing parasitic parameters or controlling the angle of the antenna. There is a lack of research on the preparation of rectangular waveguide-type microwave devices based on origami folding. The current processing methods of rectangular waveguide-type microwave devices have the characteristics of high costs and low efficiency. The use of origami to realize the conversion of planar structures to three-dimensional structures to prepare rectangular waveguide-type microwave devices could avoid these problems.

In this paper, an innovative design method based on origami for rectangular waveguide-type microwave devices is proposed, with a single-layer structure and a multi-layer structure. Polystyrene membranes and aluminum foils are applied to prepare the planar structure. Laminated processing and laser cutting are applied to prepare the planar structure of the typical origami-based three-dimensional microwave device, which is converted into a solid structure with the aid of the selective absorption of infrared light to finalize the fabrication of a typical origami-based microwave device. Three typical rectangular waveguide-type microwave devices including a rectangular waveguide, a coupler, and a power divider are fabricated and tested. The measured results of the rectangular waveguide indicate that the return loss is superior to −15 dB and that the insertion loss is superior to −0.9 dB. The cut-off frequency of the rectangular waveguide is 8.3 GHz. The results of the prepared coupler show that the return loss and isolation are better than −15.0 dB, the insertion loss is better than −1.3 dB, and the coupling degree increases from −12.8 dB to −8.9 dB in the range of 11.0 GHz to 12.0 GHz, revealing its good performance in energy coupling with a small loss. Correspondingly, the prepared power divider demonstrates the measured results that the return loss dwindles from −8.9 dB to −11.3 dB and the insertion loss of one output port is approximate to that of the remaining one and varies between −3.5 dB and −5.2 dB in the range of 10.5 GHz to 11.5 GHz. Even though there is a certain loss, the variation in the measured results is consistent with that of the simulated results. Plainly, the origami-based design method for rectangular waveguide-type microwave devices has proved to be feasible with a low cost and high efficiency.

## 2. Materials and Methods

**Figure 1:**
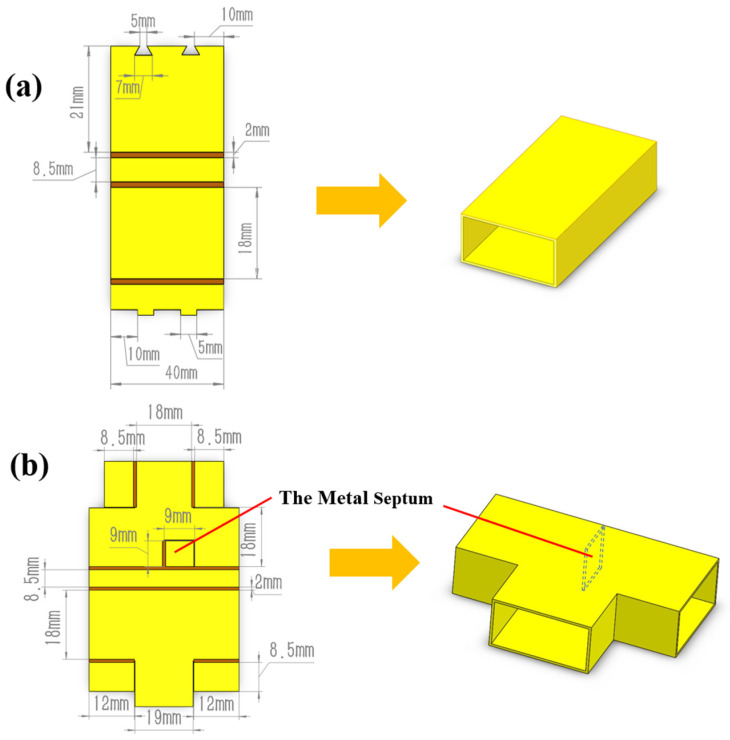

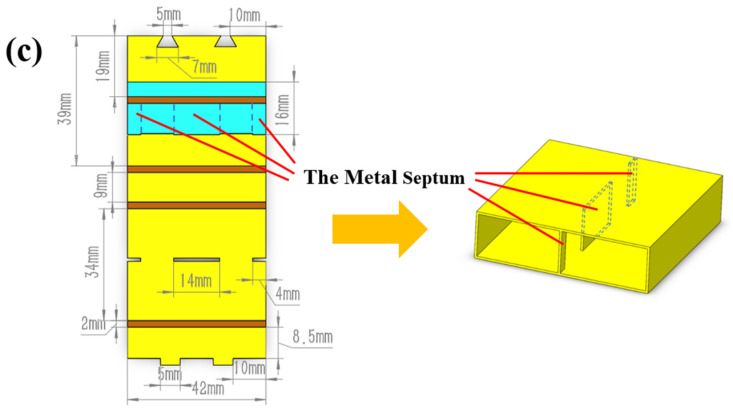
Design of the origami-based microwave devices. (**a**) Rectangular waveguide. (**b**) Power divider. (**c**) Coupler.

In order to utilize origami which can convert the planar structure into a solid structure to fabricate three-dimensional microwave devices, the specific planar structure is required to be designed with consideration of the folding sequence and folding area under the condition of heating and irradiation. Figure 1 shows the planar structures and solid structures of the origami-based rectangular waveguide, the rectangular waveguide-type power divider and the coupler. As is shown in Figure 1, for the origami-based rectangular waveguide-type power divider, the metal septum can be realized by the single-layer origami structure. However, the multi-layer origami structure is necessary for the origami-based coupler for its more complex structure. This provides a reference for the realization of the complex three-dimensional origami structure.

**Figure 2:**
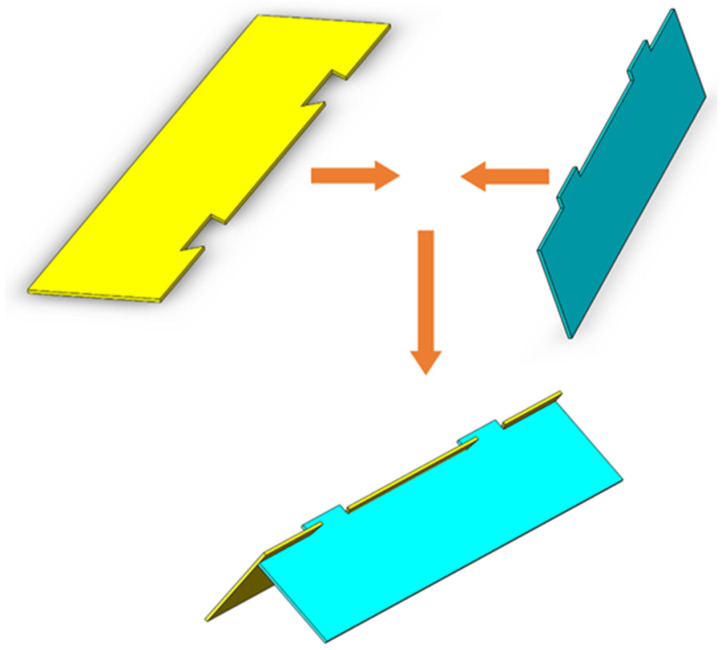
Latch-up structure. The yellow part represents a depressed structure; The blue part represents a protruding structure. The arrows indicate that the two parts are closed together.

As is shown in Figure 1a,c, the latch-up structure is introduced at the gap between the rectangular waveguide and the rectangular waveguide-type coupler to ensure the effective connection of the contact area at the closed side of the planar structure. The detailed structure is shown in Figure 2. Under the action of the latch-up structure, it can be ensured that the two waveguide walls at the gap of the rectangular waveguide are always in contact at a fixed position. At the same time, the stability of the origami structure is also assured by the latch-up structure. It should be noted that due to the complexity of the folding process of the rectangular waveguide power divider, the latch-up structure cannot be closed in the actual experiment. Therefore, the latch-up structure is not used in the preparation of the origami-based rectangular waveguide-type power divider.

Excellent stability and high folding control are essential to the supporting structure of rectangular waveguide-type devices. Here, a polystyrene membrane is adopted as the thermoactivated material to drive the origami process for the following reasons. Polystyrene is a thermoplastic material. After the heat folding is completed, it can keep its shape unchanged when the temperature is reduced, this ensures that the origami-based devices have a high stability. In addition, when the polystyrene membrane reaches a certain thickness, it has a preferable rigidity and can be used as a supporting structure material. For rectangular waveguide-type microwave devices, the use of a polystyrene membrane as the support structure does not affect the electrical and magnetic properties of the device itself. The bending process of the polystyrene membrane is relatively easy to realize and can be achieved only by local heating. Good conductivity and adhesion to the support structure are necessary for the conductive structure of rectangular waveguide-type microwave devices. Therefore, aluminum foil tape acts as a conductive structure. The material properties of the polystyrene membranes and aluminum foils are listed in Table 1 and Table 2, respectively.

**Figure 3:**
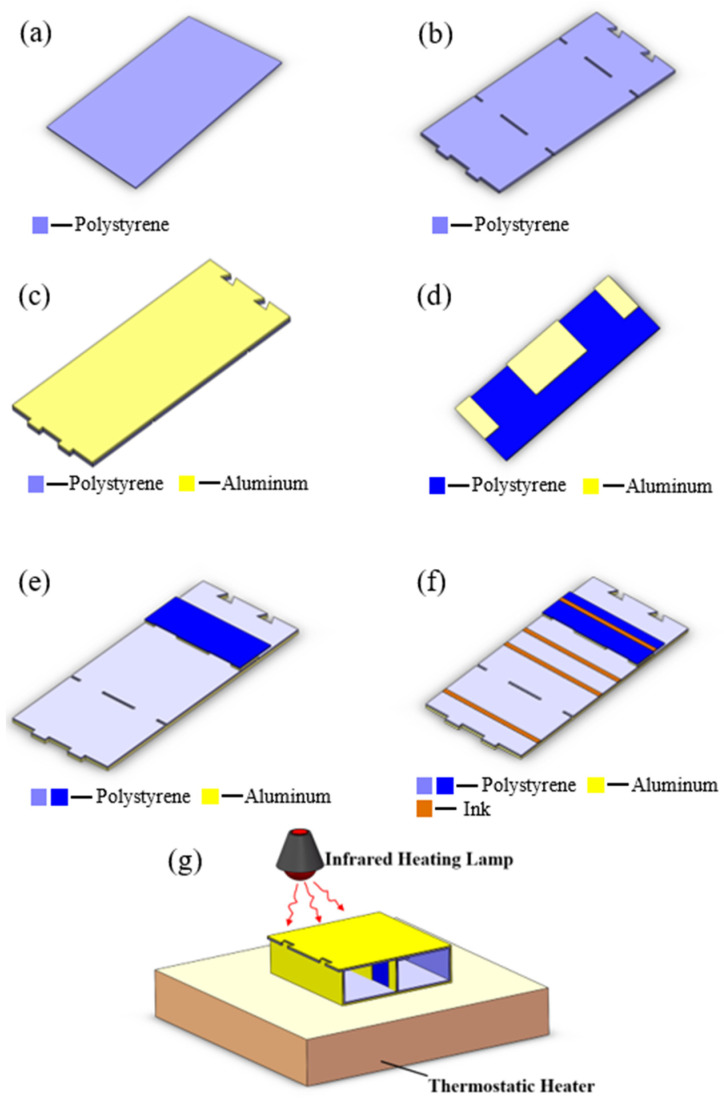
Process steps of the origami-based rectangular waveguide-type coupler. (**a**) Preparation of the polystyrene membrane. (**b**) Laser-cutting of the polystyrene membrane. (**c**) Adhesion of the aluminum foil tape. (**d**) Fabrication of the metal septum. (**e**) Lamination of the two parts by glue. (**f**) Coating of the endothermic ink on the crease area. (**g**) Converting process with irradiation of the infrared heating lamp.

Since the fabrication methods of the origami-based rectangular waveguide, waveguide-type power divider, and coupler are quite similar, the process steps of the rectangular waveguide-type coupler are taken as an example to introduce the fabrication methods. The process steps of the typical origami-based rectangular waveguide-type microwave devices are shown in Figure 3. Firstly, the polystyrene (PS) membrane is prepared as the origami material and patterned into the planar structure of an origami-based rectangular waveguide-type coupler via laser cutting: this is shown in Figure 3a,b. Then, the aluminum foil tape is attached to one side of the polystyrene membrane, realizing the metallization of the origami structure, as shown in Figure 3c. The analogous process steps are duplicated to fabricate the metal septum, which forms the coupling holes of the rectangular waveguide-type coupler: this is shown in Figure 3d. Next, the two parts are laminated together with glue to form the complete origami planar structure. The endothermic ink is coated on the crease area, as shown in Figure 3e,f. The material of the endothermic ink is Vanta black. In the end, the planar structure is converted into a solid structure with the excitation of the thermostatic heater and the irradiation of the infrared heating lamp, as shown in Figure 3g. The temperature of the thermostatic heater cannot exceed the glass transition temperature of polystyrene. The temperature in the creases rises and reaches the glass transition temperature of polystyrene. At this time, the non-creased part does not reach the glass transition temperature. Thus, the deformation of the non-creased part can be ignored compared to the crease part. The process steps of the origami-based rectangular waveguide and waveguide-type power divider are similar to those of the coupler except that the rectangular waveguide and the power divider adopt a single-layer origami structure. The metal septum, which determines the power ratio of the two output ports, is fabricated simultaneously with the waveguide wall of the power divider.

**Figure 4:**
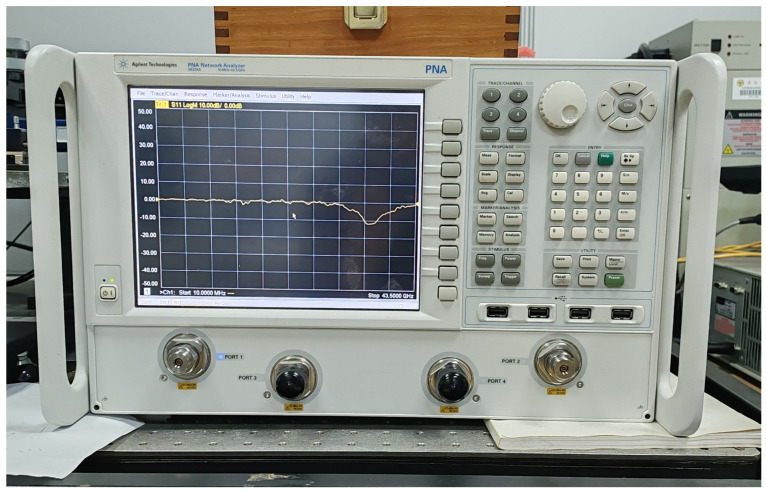
Agilent N5244A PNA-X Vector Network Analyzer.

In order to verify the feasibility of the prototype microwave devices based on the origami structure, the prepared origami-based rectangular waveguide, rectangular waveguide-type coupler, and power divider were measured using an Agilent N5244A PNA-X Vector Network Analyzer (Keysight Technologies, Santa Rosa, CA, USA), as shown in Figure 4 [33].

**Figure 5:**
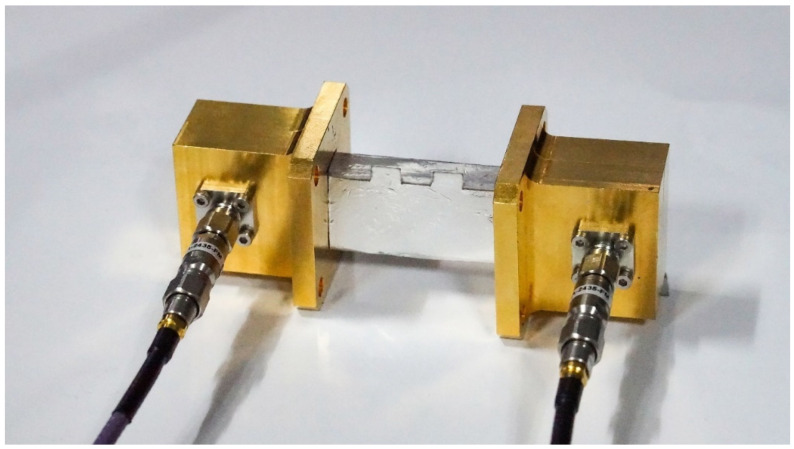
Measurement of the origami-based rectangular waveguide.

**Figure 6:**
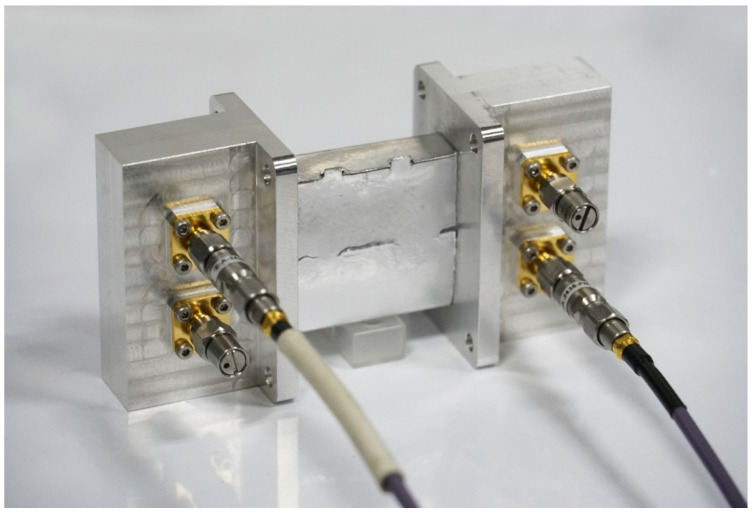
Measurement of the origami-based rectangular waveguide-type coupler.

The connection between the vector network analyzer and the origami-based rectangular waveguide is realized through the coaxial line and the adapter, which acts as a transition between the rectangular waveguide and the coaxial line, as shown in Figure 5. The adapter between the vector network analyzer and the rectangular waveguide-type power divider is the same as the adapter shown in Figure 5. For the measurement of the coupler, the required equipment and process are basically the same as those of the rectangular waveguide. However, since the coupler is a four-port and the spacing between the adjacent two ports is very small, it is essential to customize the rectangular waveguide-coaxial adapter separately. Additional 50 Ω loads are required to match the remaining ports because the vector network analyzer can only perform two-port measurements. The structure of the adapter is shown in Figure 6. Before the measurement, the instrument needs to be calibrated using the SLOT method to ensure the accuracy of the results.

## 3. Results and Discussion

### 3.1. Fabrication

Firstly, the size of waveguide-type devices is obtained according to microwave simulation. The size of the standard rectangular waveguide BJ120, which is 19 mm × 9.5 mm, is adopted as the cross-section size of waveguide-type devices. The corresponding origami plane structure style is designed, and the corresponding layout is drawn in AutoCAD 2019. The layout file is imported into the laser marking machine to cut the polystyrene film into the required structure. The power of the used layer-cutting machine is 13 W and the wavelength of the layer is 1064 nm. Then, the planar structures of typical origami-based rectangular waveguide-type microwave devices are realized by stacking processing, metal coating, patterning, and other processes. The deformation effect of the origami structure can only be significant after the crease area reaches a certain width. Here, the width of the patterned crease area is 2 mm. The thicknesses of the polystyrene membrane and aluminum foil tape are 0.3 mm and 0.06 mm, respectively. After completing the preparation of the typical origami planar structure, the thermostatic heater is used to provide the basic temperature for folding the polystyrene membrane. When the temperature of the thermostatic heater is 96.7 °C, the folding effect is the best.

**Figure 7:**
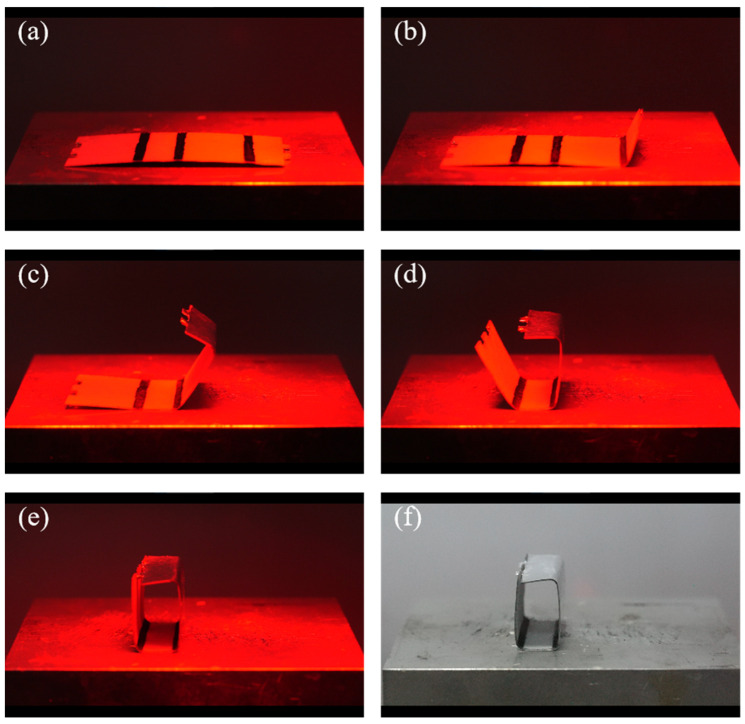
Self-folding process of the origami-based rectangular waveguide. (**a**–**e**) The folding process. (**f**) The prepared origami-based rectangular waveguide.

**Figure 8:**
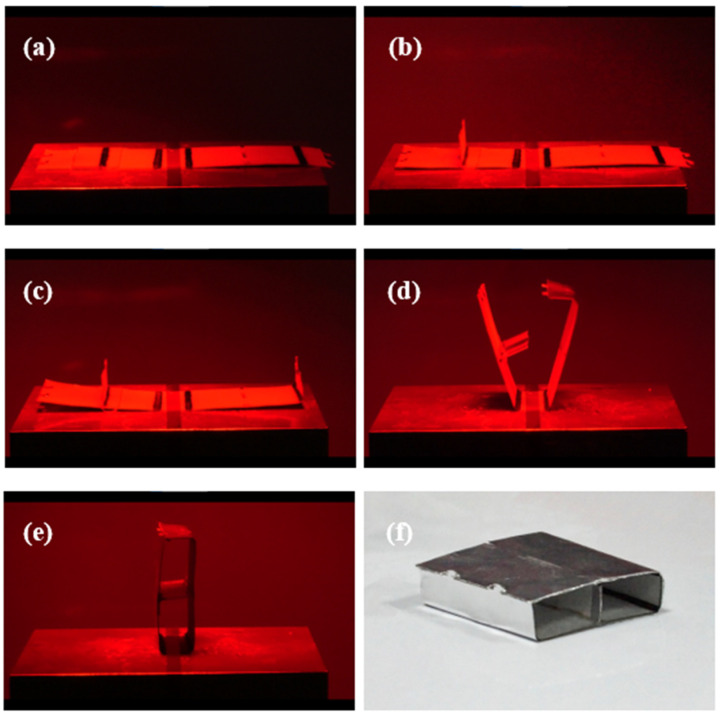
Self-folding process of the origami-based rectangular waveguide-type coupler. (**a**–**e**) The folding process. (**f**) The prepared origami-based rectangular waveguide-type divider.

**Figure 9:**
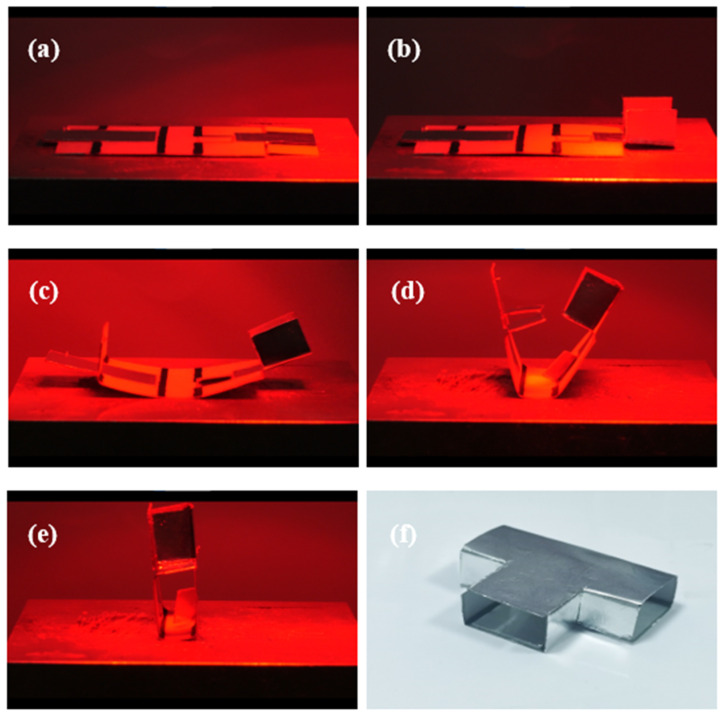
Self-folding process of the origami-based rectangular waveguide-type power divider. (**a**–**e**) The folding process. (**f**) The prepared origami-based rectangular waveguide-type divider.

The realistic self-folding processes of the rectangular waveguide, the rectangular waveguide-type coupler, and the rectangular waveguide-type power divider are shown in Figure 7, Figure 8, and Figure 9, respectively. Since the driving method used in this paper is light-driven, light occlusion cannot occur during the light-driven process. This limits the style and folding order of the origami structure. From the results shown in Figure 7, it can be seen that as the infrared heating lamp moves from right to left, the creases corresponding to the light source irradiation area are bent and folded successively. Finally the latch-up structure is successfully closed. In Figure 8, the double-layer structure of the coupler can be clearly distinguished. In the deformation process of the structure, the folding of the metal septum needs to be completed separately first; otherwise, it will be difficult to control the folding angle of multiple creases at the same time. Due to the existence of the double-layer structure, the weight of the left and right sides of the coupler is inconsistent. Therefore, the coupler could be toppled during the folding process. In order to solve this problem, the middle part of the coupler is fixed on the thermostatic heater using tape to ensure that it does not topple over. It should be noted that in the process of closing the latch-up structure, the metal septum produces a certain occlusion of light. Thus, when the two creases in the middle of the coupler are bent, it is necessary to maintain the continuous aggregation of light, so that the temperature remains high enough even if the light is occluded. Due to the latch-up structure, the prepared rectangular waveguide and the rectangular waveguide-type coupler have good closing effects. The preparation process of the rectangular waveguide-type power divider also needs to provide the initial temperature through the thermostatic heater. Then, the folding sequence of the origami plane structure is controlled by moving the position of the infrared heating lamp. The final prepared origami-based rectangular waveguide, rectangular waveguide-type coupler, and rectangular waveguide-type coupler are shown in Figure 7f, Figure 8f, and Figure 9f, respectively. For the prepared waveguide-type devices, the cross-section is slightly irregular. This irregularity is due to the fact that the crease has a width of 2 mm. The crease is bent into an arc shape by heating, which causes the cross-section of the waveguide-type devices to be irregular. This small irregularity has little effect on the performance of the device.

### 3.2. Measurements

**Figure 10:**
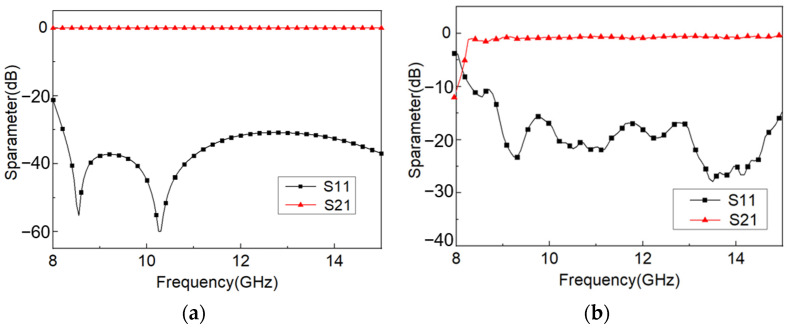
The measured and simulated results of S parameters of the origami-based rectangular waveguide: (**a**) simulated results, (**b**) measured results.

From the measured results in Figure 10b, it can be seen that the cut-off frequency of the rectangular waveguide is 8.2 GHz, which has a certain deviation from the theoretical value of 7.89 GHz. The main reason is that the creases do not always bend at the center of the black crease during the deformation process, resulting in a small deviation in the length and width of the cross-section of the rectangular waveguide, which leads to the offset of the cut-off frequency of the origami-based rectangular waveguide. The return loss of the rectangular waveguide is less than −15 dB in the frequency range of 9 GHz–15 GHz, and the insertion loss is better than −0.9 dB. The S parameters of the rectangular waveguide meet the transmission requirements of the basic transmission line in the working frequency band, and the variation law is consistent with the simulation results. There is a certain deviation between the test results and the simulated results. The main reason is that the position and length of the contact area of the origami structure deviate from the size set in the simulation due to the uncertainty of the origami angle and the crease during the actual experiment. There are also deviations in the size of the gap and the position of the contact area from the simulation, which ultimately leads to a deviation between the microwave performance results obtained by the measurement and the simulated results.

**Figure 11:**
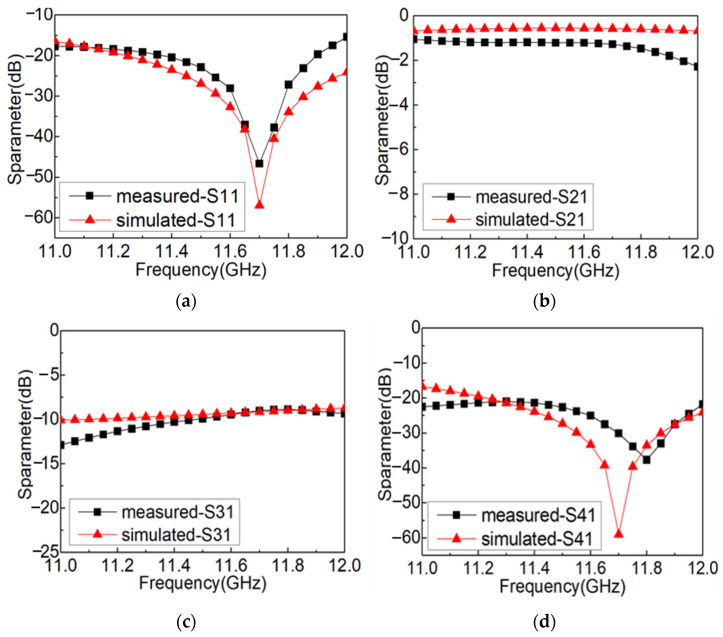
The measured and simulated results of S parameters of the origami-based rectangular waveguide-type coupler: (**a**) S11, (**b**) S21, (**c**) S31, (**d**) S41.

The measured and simulated results of the prepared coupler with the multi-layer origami structure are shown in Figure 11. As is shown in Figure 11, the measured results of the prepared coupler demonstrate that the return loss and isolation are better than −15.0 dB, the insertion loss is better than −1.3 dB, and the coupling degree increases from −12.8 dB to −8.9 dB in the range of 11.0 GHz to 12.0 GHz, which are in good accordance with the simulated results. Comparing the measured results with the simulated results, it can be seen that most of the energy is transferred to the output port through the main branch and a certain proportion of the energy is coupled to the isolation port. Comparing the simulated results with the measured results, it can be seen that there is a certain deviation between them. The main reason for the analysis is that the metal septum in the middle of the coupler has a deviation in size during the preparation process and is not in an ideal flat state during the deformation process, which leads to the deviation of the energy ratio between the main branch output port and the secondary branch coupling port. Moreover, due to the influence of the gap, the energy loss of the coupler is large, which further results in the deviation between the S parameter measured results and the simulated results of the coupler. Although there is a certain deviation between the simulation and the measurement, the energy loss induced by the structural irregularity and the crevice of the origami structure is limited, verifying the feasibility of realizing the rectangular waveguide-type coupler via origami.

**Figure 12:**
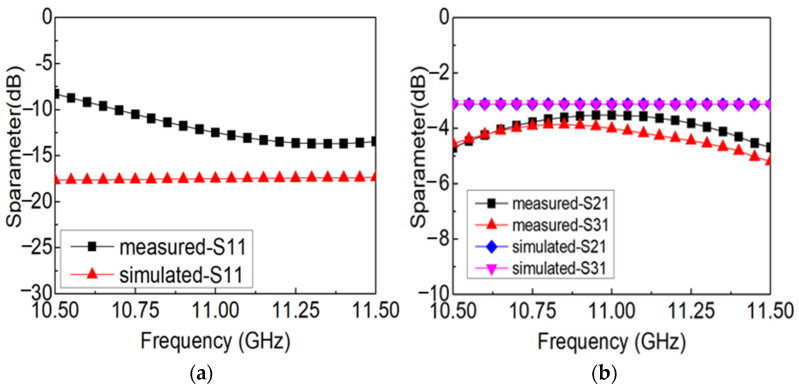
The measured and simulated results of S parameters of the origami-based rectangular waveguide-type power divider: (**a**) S11, (**b**) S21, and S31.

Correspondingly, the measured and simulated results of the prepared power divider with the single-layer origami structure are shown in Figure 12. It is demonstrated that the measured return loss dwindles from −8.9 dB to −11.3 dB and the insertion loss of one output port is approximately the same as that of the remaining one and varies between −3.5 dB and −5.2 dB in the range of 10.5 GHz to 11.5 GHz. According to the measured and simulated results, we can find that the output power of the two output ports is basically the same corresponding to the case that the metal septum is located in the middle of the power divider and the subtle difference between the output power of the two output ports is induced by the inhomogeneity of bending at the crease, making the origami structure of the power divider slightly asymmetric. Meanwhile, it can be seen that the loss of the prepared power divider is relatively larger than that of the prepared coupler, which can be explained by the reasons that the more complex structure of the power divider makes the influence of the crevice in the origami structure more obvious than the coupler. The variation law of the measured S parameters is consistent with that of the simulated results, which verifies the correctness of the origami-based design method for a rectangular waveguide-type power divider.

## 4. Conclusions

A novel origami-based design method is proposed for rectangular waveguide-type microwave devices. The solid structure can be realized easily with a low manufacturing cost and fast speed via self-folding. The rectangular waveguide, rectangular waveguide-type coupler, and power divider are fabricated with the multi-layer and single-layer origami structure, respectively. The measured results of the rectangular waveguide indicate that the insertion loss is superior to −0.9 dB. As for the prepared coupler, the return loss and isolation are better than −15.0 dB, the insertion loss is better than −1.3 dB, and the coupling degree increases from −12.8 dB to −8.9 dB in the range of 11.0 GHz to 12.0 GHz, which are consistent with the simulated results. Correspondingly, the prepared power divider demonstrates the measured results that the return loss dwindles from −8.9 dB to −11.3 dB and the insertion loss of one output port is approximate to that of the remaining one and varies between −3.5 dB and −5.2 dB in the range of 10.5 GHz to 11.5 GHz. The variation law of the measured results is in accordance with that of the simulated results, indicating the feasibility of the origami-based design method for the rectangular waveguide-type microwave devices. In future studies, selective light absorption will be tried. According to the difference in the absorption rate of different colors of inks to different colors of light, the planar structure is irradiated with different colors of light to achieve step-by-step folding. The problem of structural occlusion during illumination may be solved in this way.

## Figures and Tables

**Table 1:** Material parameters of polystyrene.

Material Parameter	Polystyrene (PS)
Thermal Conductivity/W/m·K	0.08
Relative Dielectric Constant	2.6
Electric Conductivity/S/m	$1\times 10^{-16}$
Density/kg/m^3^	1050
Young’s Modulus/GPa	3.6
Glass Transition Temperature/°C	110
Thermal Expansion Coefficient $\alpha_{1}/10^{-6}/K$	80 ^1^
$\mathrm{Thermal}\;\mathrm{Expansion}\;\mathrm{Coefficient}\;\alpha_{2}/10^{-6}/K$	10 ^2^
Specific Heat Capacity/J/kg·K	1300

^1^ Glass transition temperature when the temperature is below the glass transition temperature. ^2^ Glass transition temperature when the temperature is above the glass transition temperature.

**Table 2:** Material parameters of Aluminum.

Material Parameter	Aluminum (Al)
Electric Conductivity/S/m	$3.8\times 10^{7}$
Density/kg/m^3^	2700
Specific Heat Capacity/J/kg·K	880
Relative Magnetic Permeability	1.000021

## Data Availability

Data are available on request from the authors.
